# Schistosome Infection Intensity Is Inversely Related to Auto-Reactive Antibody Levels

**DOI:** 10.1371/journal.pone.0019149

**Published:** 2011-05-06

**Authors:** Francisca Mutapi, Natsuko Imai, Norman Nausch, Claire D. Bourke, Nadine Rujeni, Kate M. Mitchell, Nicholas Midzi, Mark E. J. Woolhouse, Rick M. Maizels, Takafira Mduluza

**Affiliations:** 1 Ashworth Laboratories, School of Biological Sciences, Institute for Immunology and Infection Research, University of Edinburgh, Edinburgh, United Kingdom; 2 National Institute of Health Research, Causeway, Harare, Zimbabwe; 3 Department of Biochemistry, University of Zimbabwe, Mount Pleasant, Harare, Zimbabwe; La Jolla Institute of Allergy and Immunology, United States of America

## Abstract

In animal experimental models, parasitic helminth infections can protect the host from auto-immune diseases. We conducted a population-scale human study investigating the relationship between helminth parasitism and auto-reactive antibodies and the subsequent effect of anti-helminthic treatment on this relationship. Levels of antinuclear antibodies (ANA) and plasma IL-10 were measured by enzyme linked immunosorbent assay in 613 Zimbabweans (aged 2–86 years) naturally exposed to the blood fluke *Schistosoma haematobium*. ANA levels were related to schistosome infection intensity and systemic IL-10 levels. All participants were offered treatment with the anti-helminthic drug praziquantel and 102 treated schoolchildren (5–16 years) were followed up 6 months post-antihelminthic treatment. ANA levels were inversely associated with current infection intensity but were independent of host age, sex and HIV status. Furthermore, after allowing for the confounding effects of schistosome infection intensity, ANA levels were inversely associated with systemic levels of IL-10. ANA levels increased significantly 6 months after anti-helminthic treatment. Our study shows that ANA levels are attenuated in helminth-infected humans and that anti-helminthic treatment of helminth-infected people can significantly increase ANA levels. The implications of these findings are relevant for understanding both the aetiology of immune disorders mediated by auto-reactive antibodies and in predicting the long-term consequences of large-scale schistosomiasis control programs.

## Introduction

Three billion people are estimated to be infected with helminths in tropical and subtropical regions. Several epidemiological studies have shown an inverse association in the prevalence of helminth infections and allergy as well as reduced allergic reactivity in individuals infected with helminth infections [1], [2], [3], [4]. Fewer studies have been conducted investigating the relationship between helminth infections and another group of immune disorders, auto-immune diseases. A global survey published in 2006 showed an inverse association between the national prevalences of the intestinal helminth *Trichuris trichiura* and multiple sclerosis [5]. The inverse relationship between helminth infections and allergy may therefore extend to auto-immunity, and is likely to have an immunological basis.

Helminths are thought to manipulate host immune responses to enhance their survival [6] and, in so doing, dampen immunopathology [7] and reduce host susceptibility to and severity of allergic [8] and auto-immune diseases [9]. Auto-immune diseases cause significant and chronic morbidity and disability [10]. Their epidemiology and aetiology remain poorly understood [10], [11], but experimental studies in mice suggest that infections with parasitic helminths such as *Schistosoma mansoni* can protect against auto-immune diseases including experimental auto-immune encephalomyelitis, the experimental model of multiple sclerosis [12], Graves hyperthyroidism [13], type 1 diabetes and experimental colitis [14], [15], [16].

All currently recognised auto-immune diseases are associated with circulating auto-reactive antibodies [17] such as anti-nuclear antibodies (ANA) and they play a central role in the diagnosis and classification of auto-immune disorders. Auto-reactive antibodies can appear long before the onset of clinical disease (e.g. anti-rheumatoid factor auto-reactive antibodies can be detected up to 14 years before the onset of rheumatoid arthritis) and detecting these antibodies in serum has been shown to have strong predictive value [11]. Despite such strong association between auto-reactive antibodies and auto-immune disease, the presence of auto-reactive antibodies and their role in biological process remains unclear [18]. Auto-reactive antibodies have been associated with other conditions including cancer, and acute tissue damage [18]. Regardless of their function, auto-reactive antibodies are believed to arise as a result of a breakdown of tolerance towards self antigens [18]. They have also been interpreted as indicators of a generally heightened immune responsiveness, itself a risk factor for auto-immune disease [19].

We conducted a study to determine the relationship between the auto-reactive anti-nuclear antibodies (ANA) and parasitic helminth infection in a human population exposed to *Schistosoma haematobium*. This was part of a long term, population-scale study of schistosome-induced systemic immuno-modulatory effects which have been suggested to modify immune responses directed against unrelated antigens such as allergens and self antigens. We designed a comparative study encompassing two villages with significantly different schistosome infection levels to determine if level of helminth endemicity affected the relationship between ANA levels and schistosome infection. Our previous studies have shown that different levels of schistosome infection intensity affect levels of schistosome-specific antibody responses [20] and experimental studies suggest that the level of helminth-induced immuno-modulation is affected by helminth infection intensity [21].

The study also determined the relationship between systemic IL-10 and ANA level. IL-10 has potent immuno-regulatory and anti-inflammatory properties (see [22]). In experimental models of human auto-immune diseases, IL-10 has been shown to mediate the prevention of autoimmune disease (reviewed in [23]). This has lead to several clinical trials using both systemically and locally introduced IL-10 as a therapeutic agent against human auto-immune diseases such as Crohn's disease [24].

Finally, the study investigated the effects of removing the helminth infection with antihelminthic drugs on ANA levels in school children (the sub-population that is the World Health Organisation's recommended target for schistosome control programmes [25]).

## Materials and Methods

### Ethical Statement

Permission to conduct the study in the region was obtained from the Provincial Medical Director and institutional and ethical approval was received from the University of Zimbabwe's Institute Review Board (UZIRB) and the Medical Research Council of Zimbabwe (MRCZ) respectively. Only compliant participants were recruited into the study and they were free to drop out at any point during the study. At the beginning of the study, participants and their parents/guardians (in case of children) had the aims and procedures of the project explained fully in the local language, Shona, and written consent was obtained from participants and parents/guardian before parasitology and blood samples were obtained. Participants are involved in an ongoing study on the effects of helminth infection on regulatory and effector immune responses. After collection of all samples, all participants were offered anti-helminthic treatment with the recommended dose of praziquantel (40 mg/kg of body weight).

### Study area

The study was conducted in two villages, Magaya and Chitate, in the Mashonaland East Province of Zimbabwe (31°90′E; 17°63′S) where *S. haematobium* is endemic. Villagers are subsistence farmers who have frequent contact with infective water (as assessed by questionnaires) due to insufficient safe water and sanitation facilities as is typical in rural Zimbabwe [26], [27]. Drinking water is collected from open wells while bathing and washing is conducted in perennial rivers surrounding the village. The study area was chosen for 3 reasons: (1) this area has not been included in the National Schistosome Control Programme and participants had not received anti-helminthic treatment for schistosomiasis or other helminth infections (assessed by questionnaire); (2) similar to most rural areas in Zimbabwe, there were no reports of soil transmitted helminths [28], [29] and our subsequent parasitological examinations confirmed this; (3) two villages inhabited by people of similar ethnic groups but with significantly difference levels of schistosome infection could be identified in close proximity to allow a comparative study as has been previously conducted in other areas of Zimbabwe [20]. The rivers in the two villages differed in their temporal patterns; those in Magaya are mostly perennial while those in Chitate are seasonal, leading to different schistosome transmission dynamics.

*Plasmodium falciparum* is the predominant species of malaria in Zimbabwe [30] where malaria transmission is largely unstable in nature and malaria transmission in the study area is classified as low and sporadic [31], [32], [33] with an annual incidence of malaria of 1-10 cases/1000 people in the area [33], [34].

### Parasitology

Stool and urine specimens were collected on three consecutive days and these were examined for *S. haematobium*, *S. mansoni* and geo-helminths using standard procedures. Briefly, urine specimens were processed by urine filtration following a standard method originally described by Mott *et al*. for diagnosis of *S. haematobium* [35]. Fresh stool specimens were processed by the Kato-Katz [36] technique and subsequently analysed by microscopy for intestinal helminths including *S. mansoni*, hookworm, *Ascaris lumbricoides* and *Trichuris trichiura*. The formol-ether concentration method was performed on a random sample of 25% of the samples to confirm results obtained by the Kato-Katz technique. After collection of all samples, all participants were offered anti-helminthic treatment with the recommended dose of praziquantel (40 mg/kg of body weight). There were no untreated controls for this study because the high levels of infection meant all compliant participants were treated as recommended by the World Health Organization's Guidelines.

### Study participants: inclusion/exclusion criteria

In order to be included in the study, participants had to meet all the following criteria: 1) have provided at least two (to allow calculation of a mean infection intensity) urine and two stool samples on consecutive days; 2) be negative for intestinal helminths including *S. mansoni* (no one was excluded on this criteria as everyone was negative for these infections); and 3) have given a blood sample for the collection of sera. A total 613 participants aged 2–86 years met these criteria and formed our study population. Of these, 369 (2–86 years old) were recruited from Chitate (low schistosome infection area) and 244 (4–86 years old) from Magaya (high schistosome infection area). Of these, 602 people gave enough sera to allow the additional IL-10 serological assay. To be included in the follow-up post-treatment study, treated participants had to: 1) be school-enrolled children (the target population for mass chemotherapy [25]) from Magaya, the high infection area, (to ensure that children with high infection levels were captured in the study) enrolled in the cross sectional study; 2) have been confirmed egg negative for schistosome infections 6 weeks post anti-helminthic treatment (no one was excluded on this basis as all children receiving ant-helminthic treatment were successfully cured of their schistosome infection); and 3) have given a blood sample for sera collection 6 months after anti-helminthic treatment (5 children were excluded on this criteria). 102 participants (5–16 years old) from Magaya were included based on these criteria.

### Laboratory assays

Auto-immune reactivity was assessed by measuring serum antibodies directed against nuclear antigens (ANA) including RNP, Sm, SSA, SSB, Scl-70, Jo-1, CENP-B, Ribosomal P, DNA and histones using a routine diagnostic ELISA kit for the detection of disease-specific anti-nuclear antibodies (REAADS ANA Test Kit, Cat 10876) in a single ELISA. According to the manufacturer's product sheet, when the kit was tested with sera from patients suffering from Systemic Lupus Erythematosus, Sjögren's Syndrome, Systemic Sclerosis and, it had a sensitivity greater than 94% for these syndromes and 95% specificity. The level of auto-immune reactivity (Units/ml (U/ml)) was calculated using negative, positive and calibrator control samples supplied by the manufacturer. HIV status was determined using the USAID-approved HIV1+2 Double Check Gold rapid serological tests (Orgenics). Positive HIV status was confirmed by a second assay using a different rapid test, Determine HIV 1/2 Ag/Ab Combo. Systemic IL-10 levels were measured by ELISA using a kit from BD Biosciences (Cat 555157) following manufacturer instructions. Participants were screened for malaria both by microscopic examination of Giemsa stained blood smears as well as by a rapid serological assay (Paracheck Pf (P. falciparum) Rapid Test (Dipstick) supplied by Orchid Biomedical Systems.

### Statistical analyses

Statistical analyses were conducted using the statistical package SPSS. The difference in schistosome infection level between the two villages was determined by χ-squared test (prevalence) and analysis of variance (ANOVA) (mean infection intensity, (log _X_ +1) transformed). The relationship between the levels of anti-nuclear antibodies ((ANA) and schistosome infection intensity before anti-helminthic treatment was determined using an ANOVA. The dependent variable was ANA level. Infection intensity categorised into 3 groups according to the severity of infection as classified by the World Health Organisation [25] as no infection (0 eggs/10 ml urine), low-mild infection (1–49 eggs/10 ml urine) and high/heavy infection (50 or more eggs/10 ml urine), sex (2 categories: male, female), village (2 categories: low infection area, high infection area), age group (3 age groups: 1 (2–10 years), 2 (11–20 years) and 3(21+ years) reflecting age groups where infection is rising, peaking and declining respectively) and HIV status (2 categories: positive, negative) were the independent variables. Age and schistosome infection intensity was categorised to satisfy the assumptions of parametric tests (see [37]). The age groups reflected the ages were schistosome infection was rising, peaking and declining. Sequential sums of squares were used with schistosome infection intensity entered last in the model to allow for the effects of all the other variables before testing for its effects.

The levels of systemic IL-10 between the two villages was compared by an ANOVA allowing for the effects of host sex, age group and schistosome infection level categorised as above. Our previous studies in villages in the same province have shown that systemic levels of IL-10 can vary with host age and schistosome infection levels [38]. Therefore, to avoid the confounding effects of host age and infection intensity on the relationship between IL-10 and ANA levels, the variation in ANA levels due to host age and infection intensity was allowed for first, in an ANOVA, together with the effects of sex, village and HIV categories as above, before determining if there was a significant association between IL-10 levels (square root transformed) and ANA levels. The effect of anti-helminthic treatment on auto-reactive antibody and on IL-10 levels was tested using a repeated measure ANOVA with age, sex and schistosome infection intensity.

## Results

### Levels of schistosome infection and ANA antibodies differ between the two villages

**Figure 1:**
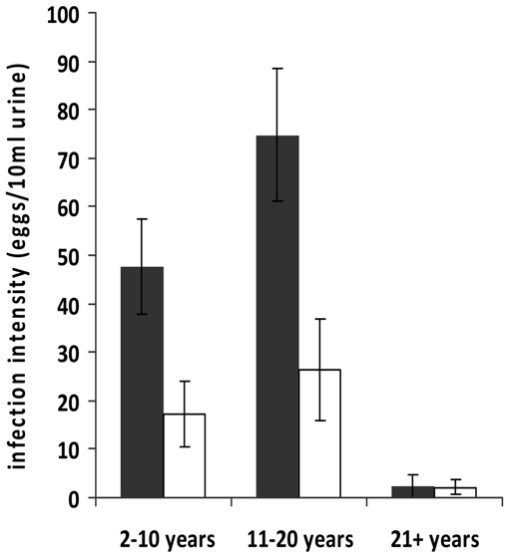
Distribution of schistosome infection intensity with age in the study villages: Mean infection intensity is higher in Magaya Village, high schistosome infection area (black) compared to Chitate Village, low schistosome infection area (white). Bars represent standard error of the mean.

We first established that the two study locations differed in levels of endemic schistosome infection. Schistosome infection prevalence was significantly higher in Magaya (prevalence  = 69%, 95% CI: 63% to 75%) than in Chitate (prevalence  = 14%, 95% CI: 11% to 18%) (χ^2^ = 187, df = 1, p<0.001), as was infection intensity 58 eggs/10 ml urine (Standard error of the mean (SEM)  = 8.02) and 15 eggs/10 ml urine (SEM  = 4.17) respectively (F_1, 612_ = 201, p<0.001). Infection levels followed the typical age-infection pattern originally described for schistosome infections with infection rising with age to peak in childhood/early adulthood before declining as shown in Figure 1. Levels of infection differed significantly between the two villages in the two younger age groups (ages 2–20years old) but were comparable in the oldest age group (21 years and above). Furthermore, the effect of interaction between age group and village of residence on schistosome infection intensities was significant (F_2,612_ = 8.96, p<0.001), indicating the phenomenon known as ‘peak shift’ [39], where peak infection levels are higher and occur earlier in areas of high schistosome transmission compared to low schistosome transmission areas. Given the significant differences in schistosome infection level in the two villages, we determined if mean ANA levels differed between the two villages. This was the case (F_1,612_ = 57,6 p<0.001) with the mean ANA level of 22.6 U/ml (SEM = 1.13) in Chitate, the low infection area, being significantly higher than the ANA level of 17 U/ml (SEM = 1.30) in Magaya, the high infection area.

**Figure 2:**
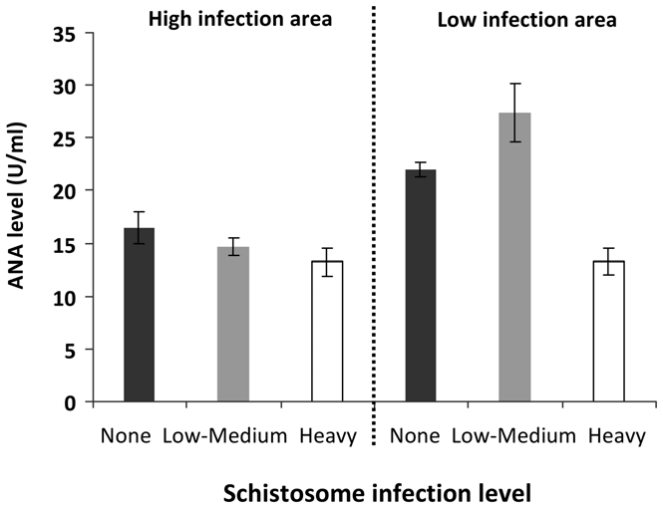
Levels of anti-nuclear antibody (ANA) levels in the different infection groups in the study villages. Bars represent individuals carrying no infection in black, low-mild infection intensity (1–49 eggs/10 ml urine) in grey and high infection intensity (50+ eggs/10 ml urine) in white. *S. haematobium* infection intensity was quantified by urine filtration. Error bars represent standard error of the mean.

When the two study populations were partitioned by the levels of schistosome infection recommended by WHO (Table 1), there were no significant differences in the mean infection intensity in the uninfected category (0 egg/10 ml urine) and in the heavy infection category (50+ eggs/10 ml urine). However, in the low-mild infection category (1–49 eggs/10 ml urine), mean infection intensity was higher in the high infection area compared to the low infection area (mean of 18 eggs vs. 15eggs/10 ml urine, F_1, 145_ = 5,4, p< = 0.022). ANA levels are shown for each infection group for the two villages in Figure 2.

**Table 1:** Description of study populations' infection levels.

Residential area	WHO Infection code	Sample size	Mean schistosome infection intensity	Standard error of the mean
**High infection area**	None	76	0	0.00
	Low-Medium	115	18	1.23
	High	53	226	26.03
	Total	244	58	8.02
**Low infection area**	None	316	0	0.00
	Low-Medium	31	10	1.72
	High	22	242	49.51
	Total	369	15	4.15
**Total study population**	None	392	0	0.00
	Low-Medium	146	16	1.07
	High	75	230	23.26
	Total	613	32	4.13

Table shows sample sizes of the number of people in each infection level in the two areas. The infection levels are based on WHO classification of *Schistosoma haematobium* infection into no infection (0eggs/10 ml urine), low-medium infection (1–49 eggs/10 ml urine) and heavy infection (50+ eggs/10 ml urine) [25].

### Anti-nuclear antibody levels are inversely related to schistosome infection intensity in the individuals

**Figure 3:**
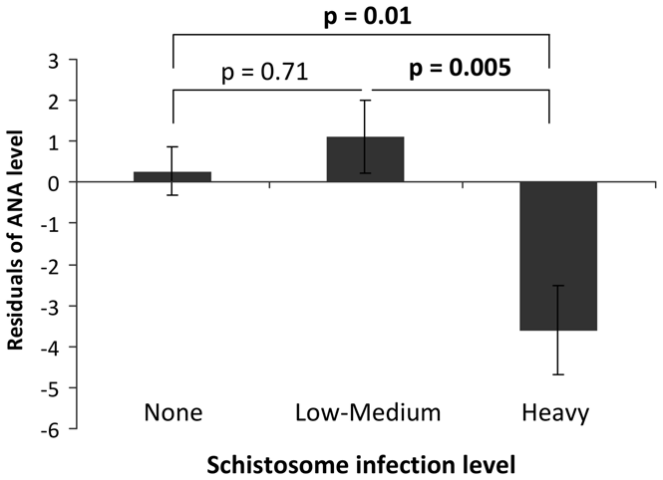
Relationship between anti-nuclear antibody (ANA) and schistosome infection levels. Bars represent residual variation in ANA levels after allowing for the variation due to host sex, age group, HIV status and village.

**Figure 4:**
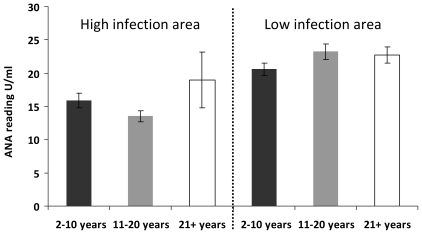
Relationship between anti-nuclear antibody (ANA) levels and host age in the study villages. Error bars represent standard error of the mean.

We tested for the relationship between ANA level and schistosome infection intensity. After allowing for the variation due to age group, sex, HIV status and place of residence, the analysis showed that levels of ANA in the two villages were inversely related to individuals' schistosome infection level (F_1,612_ = 4.3, p<0.05), with significant differences occurring between people with no infection or low-medium infection and those carrying heavy infections (Figure 3). This analysis also showed that participant age (divided into 3 age groups) did not have a significant effect on ANA levels (F_2,612_ = 0.49, p = 0.61), Figure 4 and neither did HIV status (F_1,612_ = 2.9, p = 0.09) nor participant sex (F_1,612_ = 1.22, p = 0.26).

### Anti-nuclear antibody levels increase following anti-helminthic treatment

**Figure 5:**
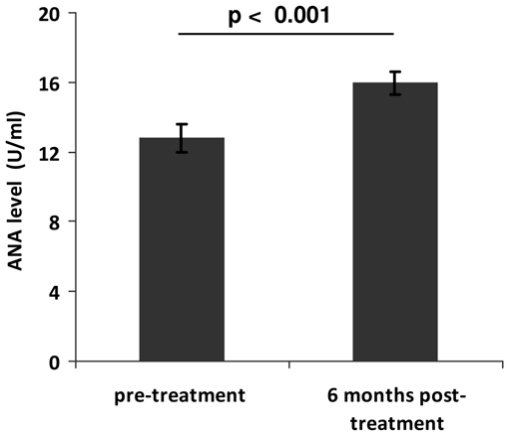
Effect of antihelminthic treatment on anti-nuclear antibody (ANA) levels. Increase in of ANA levels in school children from the high schistosome infection area, Magaya six months after anti-helminthic treatment with praziquantel.

To address the question of whether schistosome infection may be causing a reduction of ANA levels in infected subjects, we examined a cohort of 102 school children aged 5–16 years of age from the high infection area, Magaya, 6 months following treatment with the anti-helminthic praziquantel to remove adult schistosome parasites. Before treatment schistosome infection prevalence was 75% amongst these children (77/102) and infection ranged from 0–800 eggs/10 ml urine with a mean of 62 eggs/10 ml urine and SEM of 12,36. The treatment efficacy check at six weeks post-treatment showed that all 102 were egg negative for schistosome infection. By six months after treatment just 11 of the children had become re-infected with schistosomes, and with only low intensities of infection (range from 0–48 eggs/10 ml urine with a mean of 1 egg/10 ml urine and SEM of 0.50. Furthermore, at the six month post-treatment time point, auto-reactive antibody levels had risen significantly (F_1, 99_ = 15. 85, p<0.001) as shown in Figure 5.

### Anti-nuclear antibody levels are inversely related to systemic IL-10 levels

**Figure 6:**
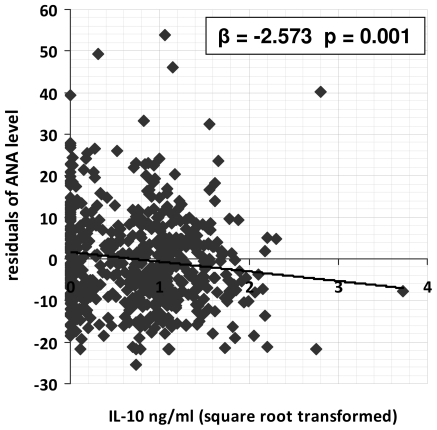
Relationship between anti-nuclear antibody (ANA level) and systemic IL-10 levels. The relationship between ANA (residual variation in ANA levels after allowing for the variation due to host sex, age group, HIV status and village) and IL-10 levels (square root transformed). P value is derived from ANOVA.

Systemic IL-10 levels in Chitate were less than those in Magaya at 0.802 ng/ml (SEM 0.073) compared to 1.07 ng/ml (SEM = 0.049), this difference was statistically significant (F_1,602_ = 50.0, p<0.001) after allowing for host age, sex, age group, ANA level and schistosome infection level. In both villages schistosome infection was negatively associated with IL-10 levels (Chitate F_2,365_ = 5.12, p = 0.006; Magaya F_2, 237_ = 2.70, p = 0.069). In the high infection area the relationship between infection level and schistosome infection changed with host age (F_2, 237_ = 6.34, p = 0.002) which was not the case in the low infection are (F_2, 365_ = 2.84 p = 0.06). Furthermore, ANA levels were inversely associated with IL-10 levels (F_1,602_ = 10.3, p = 0.01) after allowing for the effects of residential area, host age, sex, age group and schistosome infection level as shown in Figure 6. Following treatment, 78 of the 102 children gave enough serum to also measure systemic IL-10 levels, and analyses of these results showed that IL-10 levels rose significantly following treatment (F_1,74_ = 200, p<0.001).

## Discussion

This study investigated the distribution of anti-nuclear antibodies in human populations naturally exposed to a helminth infection. Our results showed that ANA levels were significantly lower in a population exposed to higher levels of *S. haematobium* infection compared to a population exposed to lower levels. Moreover, within those populations ANA levels were negatively correlated with schistosome infection level. This relationship was robust to potential confounders, host age, sex, HIV status and residential location. In school-aged children, ANA levels increased following curative anti-helminthic treatment with praziquantel.

None of the participants in this study were positive for malaria infection (both by microscopic examination of blood smear or serological detection of *Plasmodium falciparum* antigens); therefore ANA levels in these populations cannot be attributed to malaria infection as has been reported in other settings [40]. However, there are several possible explanations for an association between infectious agents and ANA levels. First, the molecular mimicry hypothesis has been suggested to explain a relationship between some pathogens and ANA levels as well as auto-immune reactivity, i.e. antigenic determinants of micro-organisms may resemble antigenic determinants of their host, and so elicit an auto-immune response that harms the host [41]. The prediction of this hypothesis would be a positive association between schistosome infection and ANA levels which contradicts the observation in this study. Furthermore, removal of the pathogen by treatment would not lead to an increase in ANA levels. Second, the drug praziquantel could, in theory, increase auto-reactive antibody levels. Some drugs can induce auto-reactive antibodies which would subsequently disappear after stopping drug therapy [42]. However, there have not been any reports of praziquantel inducing ANA and, furthermore, drug induced auto-reactivity would not explain the inverse association between ANA and schistosome infection level before treatment, since all participants had no prior history of anti-helminthic treatment. Third, ANA could be protective against schistosome infection, so that the ANA production preceded exposure to infection and provided protection against infection. This explanation has been proposed in malaria infections (reviewed in [43]) where cross-reactivity occurs between human and *Plasmodium falciparum* antigens and correlates with clinical protection against malaria [44]. While this hypothesis might explain the negative association between schistosome infection and ANA levels, it again, does not explain the rise in ANA levels following anti-helminthic treatment. Furthermore, it does not explain the difference in ANA levels between the two areas (both populated by the same ethnic group) with different levels of schistosome infection in the two populations.

We consider that the best explanation for the negative association between schistosome infection and ANA levels observed in our study is the so-called ‘hygiene hypothesis’ first proposed by Strachan in 1989 [45]. The hypothesis suggests that exposure to infectious agents, such as parasitic helminths, elicits immune deviation or dampening that serves to reduce incidence or severity of allergy [8] and auto-immune reactivity [9]. This may result from the need for helminths to manipulate host immune responses to enhance their survival [7]. One prediction from this hypothesis is that schistosome infection levels will be inversely associated with ANA levels. Furthermore, in keeping with results from experimental allergy studies [21], the higher the overall schistosome infection level, the lower the expected level of ANA reactivity in the population. This hypothesis would also predict that removing the schistosome worms would result in an increase in ANA levels. Our results are consistent with all these predictions. To date, there are no published studies on the effects of anti-helminth treatment on ANA levels in human or experimental studies. The increase in ANA levels we report is consistent with reports from the allergy studies which show an increase in atopic reactivity following anti-helminthic treatment [46].

Our demonstration that systemic IL-10 levels were significantly higher in Magaya (the high infection areas) and that they were inversely related to auto-reactive antibody levels in both villages is consistent with IL-10 being associated with immuno-modulation of host responses.[22]. The overall increase in systemic IL-10 levels following treatment may be related to an increase in schistosome-specific IL-10, as has been reported in human *S. mansoni* infections [47]. A possible explanation for this increase may be that the parasite antigens presented to the immune system as a result of dying schistosome worms may induce an increase in IL-10 levels.

Our results show a clear relationship between schistosome infection levels and ANA levels auto-reactive antibody levels. ANA levels were reduced in helminth-infected humans and anti-helminth treatment of helminth-infected people resulted in a significant increase in ANA levels. The results indicate the need for further investigation into the nature and aetiology of the association between helminth infections and ANA. Furthermore, there is need for further studies on the implications of high ANA levels in terms of the development of pathology. The use of population-based studies with different levels of helminth endemicity such as ours, complemented by experimental mechanistic studies in animal models will clarify the relationship between the epidemiology/aetiology of immune disorders and different helminth groups pre- and post anti-helminth treatment. Our study adds to the growing studies characterising the short-term and long-term effects of antihelminthic treatment [48] in the face of current large scale global helminth control initiatives whose long-term health consequences have yet to be fully understood.
